# Burden of illness and mortality in men with Adrenomyeloneuropathy: a retrospective cohort study

**DOI:** 10.1186/s13023-024-03276-w

**Published:** 2024-07-17

**Authors:** Joshua L. Bonkowsky, Bridget Healey, Naomi C. Sacks, Ronaé McLin, Philip L. Cyr, Eileen K. Sawyer, Christopher D. Stephen, Florian Eichler

**Affiliations:** 1 Primary Children’s Hospital, Intermountain Healthcare, Salt Lake City, UT USA; 2 PRECISION AQ, 133 Federal St.,10th Floor, Boston, MA 02110 USA; 3 Ontada, Boston, MA USA; 4 HEORStrategies, A Division of ToxStrategies Inc, Boston, MA USA; 5 SwanBio Therapeutics, Bala Cynwyd, PA USA; 6 Department of Neurology, Massachusetts General Hospital, Harvard Medical School, Boston, MA USA; 7 Department of Pediatrics, University of Utah School of Medicine, Salt Lake City, UT USA

## Abstract

**Background:**

Adrenomyeloneuropathy (AMN) is a neurodegenerative disease phenotype of X-linked adrenoleukodystrophy (ALD), resulting in progressive myeloneuropathy causing spastic paraparesis, sensory ataxia, and bowel/bladder symptoms. We conducted a retrospective cohort study using two large administrative databases to characterize mortality and the burden of illness in adult men with AMN in the US.

**Results:**

Healthcare resource use was assessed using a national commercial insurance claims database (2006–2021). Males with AMN ages 18–64 years and no evidence of cerebral ALD or other peroxisomal disorders were included and 1:4 matched on demographic characteristics to individuals without AMN. All study participants were followed for as long as observable. Patients with AMN were also identified in the Medicare Limited Dataset (2017–2022); mortality and age at death were compared with all Medicare enrollees. We identified 303 commercially insured men with AMN. Compared with non-AMN, individuals with AMN had significantly more inpatient hospital admissions (0.44 vs. 0.04 admissions/patient/year), outpatient clinic (8.88 vs. 4.1 visits/patient/year), outpatient hospital (5.33 vs. 0.99 visits/patient/year), and home healthcare visits (4.66 vs. 0.2 visits/patient/year), durable medical equipment claims (0.7 vs. 0.1 claims/patient/year), and prescription medication fills (18.1 vs. 5.4 fills/patient/year) (all *p* < 0.001). Average length-of-stay per hospitalization was also longer in AMN (8.88 vs. 4.3 days; *p* < 0.001). Rates of comorbidities were significantly more common in AMN compared to controls, including peripheral vascular disease (4.6% vs. 0.99%), chronic pulmonary disease (6.3% vs. 2.6%), and liver disease (5.6% vs. 0.88%), all *p* < 0.001. Among individuals age < 65 with Medicare disability coverage, mortality rates were 5.3x higher for adult AMN males (39.3% vs. 7.4%) and the age at death significantly younger (47.0 ± 11.3 vs. 56.5 ± 7.8 years), both *p* < 0.001. Among Medicare beneficiaries ages ≥ 65 mortality rates were 2.2x higher for men with AMN vs. those without AMN (48.6% vs. 22.4%), *p* < 0.001.

**Conclusion:**

AMN imposes a substantial and underrecognized health burden on men, with higher healthcare utilization, greater medical comorbidity, higher mortality rates, and younger age at death.

**Supplementary Information:**

The online version contains supplementary material available at 10.1186/s13023-024-03276-w.

## Introduction

### Background

X-linked adrenoleukodystrophy (X-ALD) is a neurogenetic condition affecting children and adults with an incidence of 1:16,800, caused by a mutation of the *ABCD1* gene on the X chromosome [1, 2]. Nearly all males with the mutation who reach adulthood develop adrenomyeloneuropathy (AMN) [3]. AMN is characterized by a chronic progressive axonopathy affecting sensory ascending and motor descending spinal cord tracts, leading to progressive spastic paraparesis, peripheral neuropathy, ataxia, sphincter incontinence, and sexual dysfunction, sometimes accompanied by adrenal insufficiency [3–6]. 

There are currently no effective treatments for preventing, stabilizing, or reversing AMN progression. Moreover, care must be individualized, given clinical heterogeneity [7]. In its early stages, AMN can present with neuropathic symptoms, typically treated with analgesics, anti-spasmodics, and botulinum toxins, as well as with bladder symptoms, managed initially with lifestyle changes [8]. When adrenal insufficiency is identified, corticosteroid replacement therapy is essential and can be lifesaving [9]. AMN disease progression often is marked by muscle spasms and walking difficulties. Physical therapy (PT), management of urologic complications, and family and/or vocational counseling are frequently used [9]. In advanced AMN, when walking is severely impaired, patients rely on durable medical equipment (DME), such as walkers or wheelchairs.

Despite its debilitating nature, little is known about the direct medical burden of AMN, accompanying morbidities, or its effect on mortality. In this study of US adult men with AMN, the primary objectives were to: (1) characterize the demographic and clinical characteristics; (2) quantify healthcare resource utilization (HRU) and medical costs to assess medical burden; and (3) assess mortality.

## Methods

### Study design and data sources

We conducted a retrospective cohort study using United States (US) health insurance claims, demographic, and enrollment data. Two data sources were used. IQVIA’s PharMetrics Plus database (1/1/2006-6/30/2021) was used to determine patients with AMN demographic and clinical characteristics, all-cause HRU, and the actual amounts paid by payers for all-cause medical and prescription medication services. The PharMetrics Plus data is representative of the US commercially insured population for individuals < 65 years and contains demographic, enrollment, and fully adjudicated medical and prescription drug claims data for approximately 150 million deidentified individuals enrolled in US commercial health insurance plans, with an annual capture of approximately 40 million individuals [10]. 

PharMetrics Plus data do not, however, include mortality. Consequently, we used the Medicare Limited Data Set (LDS) (1/1/2016-12/31/2020) to examine mortality among patients with AMN. The LDS contains enrollment, demographic and claims data for deidentified Medicare beneficiaries, with approximately 60 million Medicare beneficiaries enrolled in or entitled to Medicare, within a given calendar year. These beneficiaries include age-eligible individuals (≥ 65 years) and individuals with Medicare coverage who are disability-eligible or eligible related to end-stage renal disease (< 65 years) [11]. Medicare claims data from the Medicare Common Working File, online date of death edits submitted by the deceased’s family members, and benefit information collected from the Railroad Retirement Board and the Social Security Administration are the main sources used to develop LDS mortality data – the Master Beneficiary Summary File base segment within LDS contain date of death but not cause of death [12]. 

### Study patients

**Fig. 1:**
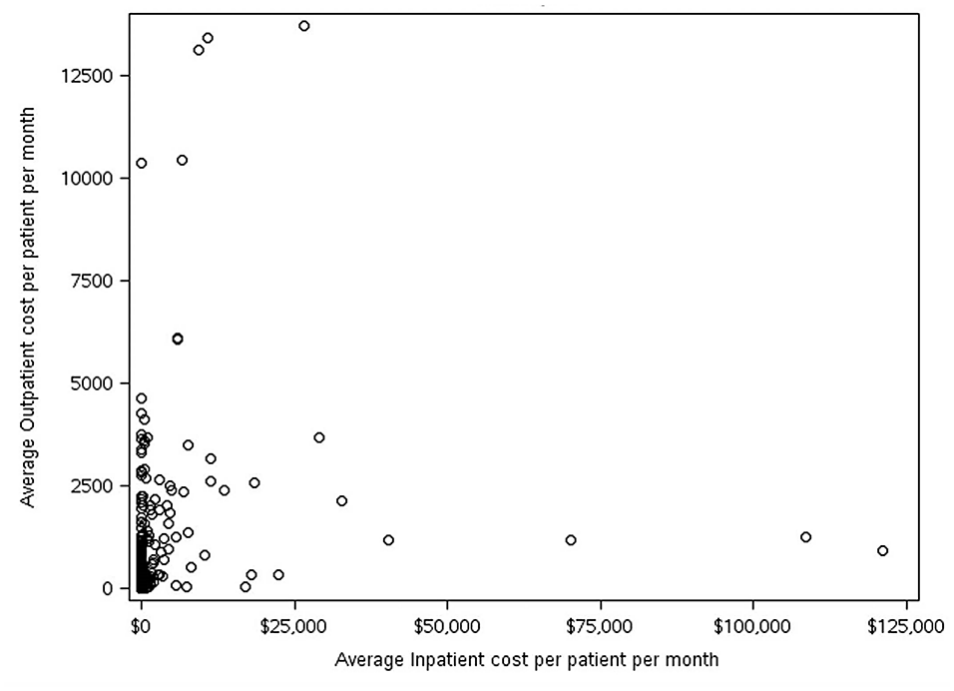
Average in-patient and out-patient costs per month for patients meeting study criteria

**Fig. 2:**
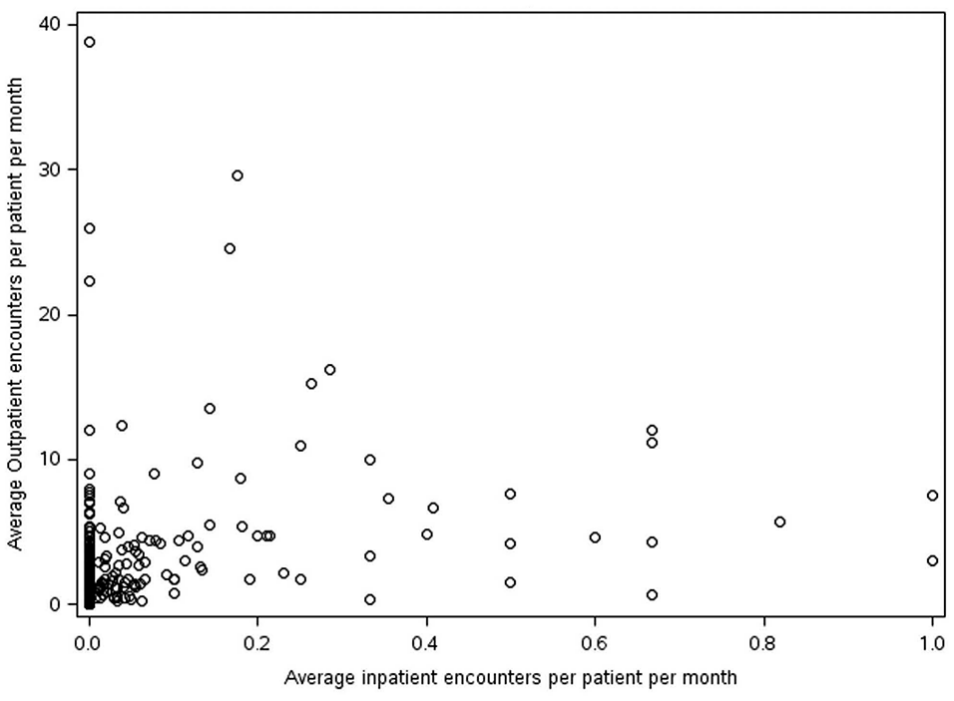
Average in-patient and out-patient healthcare encounters per month for patients meeting study criteria

Study patients were male adults with ≥ 1 inpatient or ≥ 2 outpatient claims with a diagnosis of AMN (International Classification of Diseases, 9th or 10th revision, Clinical Modification (ICD-9-CM/ICD-10-CM) claim (Table 1). Individuals with evidence of Zellweger syndrome (ICD-10-CM: E71.510), rhizomelic chondrodysplasia punctata (ICD-10-CM: E71.540), and/or childhood cerebral X-linked adrenoleukodystrophy (ICD-10-CM: E71.520) were excluded. There is no ICD diagnosis code for adult cerebral adrenoleukodystrophy, which may be associated with very high costs. Consequently, we examined the distribution of outpatient utilization and costs relative to inpatient utilization and costs. However, we did not observe any association that might lead to stratifying patients with adult cerebral adrenoleukodystrophy (Figs. 1 and 2). Commercially insured individuals were limited to those age 18–64, while those with Medicare coverage were age 18 and above, with disability-eligible beneficiaries age < 65, and age-eligible age ≥ 65. Inclusion and exclusion criteria were applied to study patients with commercial and Medicare insurance coverage.

**Table 1:** ICD-CM-# ALD + AMN diagnosis codes

Code Type	Code	Description
ICD-9-CM	27,786	Peroxisomal disorders
ICD-10-CM	E71521	Adolescent X-linked adrenoleukodystrophy
	E71522	Adrenomyeloneuropathy
	E71528	Other X-linked adrenoleukodystrophy
	E71529	X-linked adrenoleukodystrophy, unspecified type

Commercially insured study patients were 1:4 propensity score matched (PSM) to individuals with no evidence of AMN on the basis of sex, age, geographic region, and continuous enrollment time period. To permit assessment of differences in coexisting clinical conditions, these non-AMN controls were not matched to AMN cases based on comorbid conditions.

All patients were followed from their index date, which was defined as the date of the first claim with a qualifying diagnosis in the study data. Commercially insured patients were observed for as long as they were enrolled and observable and censored when they were lost to follow-up. For measuring mortality, study patients with Medicare coverage were followed until they were no longer enrolled in Medicare fee-for-service coverage or died.

### Study measures

Demographic characteristics (gender, age, and geographic region) were identified using demographic information in the PharMetrics Plus and LDS data. Patient demographic characteristics were measured as of the index date. Patient comorbidities and clinical characteristics, as well as HRU and costs, were measured in the post-index observation window.

Individual’s comorbid conditions were identified using clinical codes recorded on claims (Tables 2 and 3). In addition, the Charlson Comorbidity Index [CCI], a commonly used measure of health status was calculated. The CCI examines and sums comorbid conditions associated with an increased likelihood of mortality. Higher CCI scores are associated with poorer health status and an increased risk of death [13, 14].

**Table 2:** Charlson comorbidity diagnosis codes

Type	Description	ICD-9 CM Code(s)	ICD-10 CM Code(s)
Charlson Comorbidity	Myocardial Infarction	410.*, 412.*	I21.*, I22.*, I25.2
	Congestive Heart Failure	428.*	I09.9, I11.0, I13.0, I13.2, I25.5, I42.0, I42.5–I42.9, I43.x, I50.x, P29.0
	Peripheral Vascular Disease	443.9, 441.*, 785.4, V43.4 Procedure 38.48	I70.*, I71.*, I73.1, I73.8, I73.9, I77.1, I79.0, I79.2, K55.1, K55.8, K55.9, Z95.8, Z95.9
	Cerebrovascular Disease	430.*–438.*	G45.*, G46.*, H34.0, I60.*–I69.*
	Dementia	290.*	F00.*–F03.*, F05.1, G30.*, G31.1
	Chronic Pulmonary Disease	490.*–505.*, 506.4	I27.8, I27.9, J40.*–J47.*, J60.*–J67.*, J68.4, J70.1, J70.3
	Rheumatic Disease	710.0, 710.1, 710.4, 714.0–714.2, 714.81, 725.*	M05.*, M06.*, M31.5, M32.*–M34.*, M35.1, M35.3, M36.0
	Peptic Ulcer Disease	531.*–534.*	K25.*–K28.*
	Liver Disease	571.2, 571.4–571.6, 456.0–456.21, 572.2–572.8	B18.*, K70.0–K70.3, K70.9, K71.3–K71.5, K71.7, K73.*, K74.*, K76.0, K76.2–K76.4, K76.8, K76.9, Z94.4, I85.0, I85.9, I86.4, I98.2, K70.4, K71.1, K72.1, K72.9, K76.5, K76.6, K76.7
	Diabetes	250.0–250.7	E10-E14
	Hemiplegia or Paraplegia	344.1, 342.*	G04.1, G11.4, G80.1, G80.2, G81.*, G82.*, G83.0–G83.4, G83.9
	Renal Disease	582.*, 583–583.7, 585.*, 586.*, 588.*	I12.0, I13.1, N03.2–N03.7, N05.2– N05.7, N18.*, N19.*, N25.0, Z49.0– Z49.2, Z94.0, Z99.2
	Cancer	140.*–172.*, 174.*.–195.8, 200.*–208.*, 196.*–199.1	C00.*–C26.*, C30.*–C34.*, C37.*– C41.*, C43.*, C45.*–C58.*, C60.*– C76.*, C81.*–C85.*, C88.*, C90.*–C97.*, C77.*–C80.*
	HIV/AIDS	042.*–044.*	B20.*–B22.*, B24.*

**Table 3:** Neuropathy diagnosis codes

Condition	Code type	Code	Description
Diabetic Neuropathy	ICD-9-CM	249.6X	Secondary diabetes mellitus with neurological manifestation
		250.6X	Diabetes with neurological manifestations
	ICD-10-CM	E13.4X	Other specified diabetes mellitus with neurological complications
		E11.4X	Type 2 diabetes mellitus with neurological complications
		E10.4X	Type 1 diabetes mellitus with neurological complications
		E09.4X	Drug or chemical induced diabetes mellitus with neurological complications
		E08.4X	Diabetes mellitus due to underlying condition with neurological complications
Non-diabetic neuropathy	ICD-9-CM	337.0X	Idiopathic peripheral autonomic neuropathy
		354.X	Mononeuritis of upper limb and mononeuritis multiplex
		355.X	Mononeuritis of lower limb and unspecified site
		356.X	Hereditary and idiopathic peripheral neuropathy
		357.X	Inflammatory and toxic neuropathy
	ICD-10-CM	G56.X	Mononeuropathies of upper limb
		G57.X	Mononeuropathies of lower limb
		G58.X	Other mononeuropathies
		G60.X	Hereditary and idiopathic neuropathy
		G61.X	Inflammatory polyneuropathy
		G62.X	Other and unspecified polyneuropathies
		G63	Polyneuropathy in disease classified elsewhere
		G90.0X	Idiopathic peripheral autonomic neuropathy

Healthcare resource use was identified by examining claims for inpatient admissions, outpatient encounters, and prescription medications for each patient. Setting-level information was identified using the place-of-service recorded on each patient’s medical claims. Inpatient admissions were measured overall and stratified by those with and those without an intensive care unit (ICU) admission. Outpatient encounters included emergency department (ED), outpatient hospital, office, laboratory and imaging testing, home healthcare, and physical therapy visits, durable medical equipment, and other outpatient encounters. ED visits were those where a patient was not hospitalized, as ED visits that result in a hospitalization are rolled into the claim for the hospital admission. Prescription drug information was evaluated using information on claims for filled prescriptions and prescription medications for AMN symptomatic treatment (including adrenal insufficiency, mood disorders, neuropathy, incontinence, and sexual dysfunction) identified using Generic Product Identifier (GPI) codes.

Costs (pharmacy, inpatient, and outpatient costs) were identified using the actual amounts paid by health plans to providers, which were recorded on each claim.

Analyses of mortality were conducted using Medicare LDS data. Patients with AMN were compared to the overall population of Medicare enrollees during the Medicare study window (1/1/2016-12/31/2020). Age-eligible (age ≥ 65) and disability-eligible (age < 65) beneficiaries were evaluated separately.

### Statistical analysis

Univariate analyses were conducted and unadjusted counts for study patients reported. Because adults with AMN might move from commercial to other commercial insurance plans or public insurance coverage, or die, we followed each patient as long as they were observable in the data and calculated HRU rates and costs over the time they were observable [15]. HRU and costs, were then estimated as averages per patient per year (PPPY), included individuals without utilization (0 encounters) or with no costs ($0 USD) recorded for a given healthcare encounter.

Bivariate analyses, Mann-Whitney U/Student’s t-test for continuous variables and Fisher’s Exact/Chi-squared tests for discrete variables, were used to compare unadjusted counts to assess differences between cases and controls.

Mortality outcomes, based on Medicare data, were calculated as the crude death rate and average over the 5-year study window.

Statistical analyses were conducted using SAS 9.3 (SAS Institute, Cary, NC) and R 2022.02. Statistical significance was defined as a two-tailed *p* < 0.05.

### Ethics

This study was exempt from Institutional Review Board approval, as it involved secondary data analyses of fully deidentified data.

## Results

### Patient characteristics

**Fig. 3:**
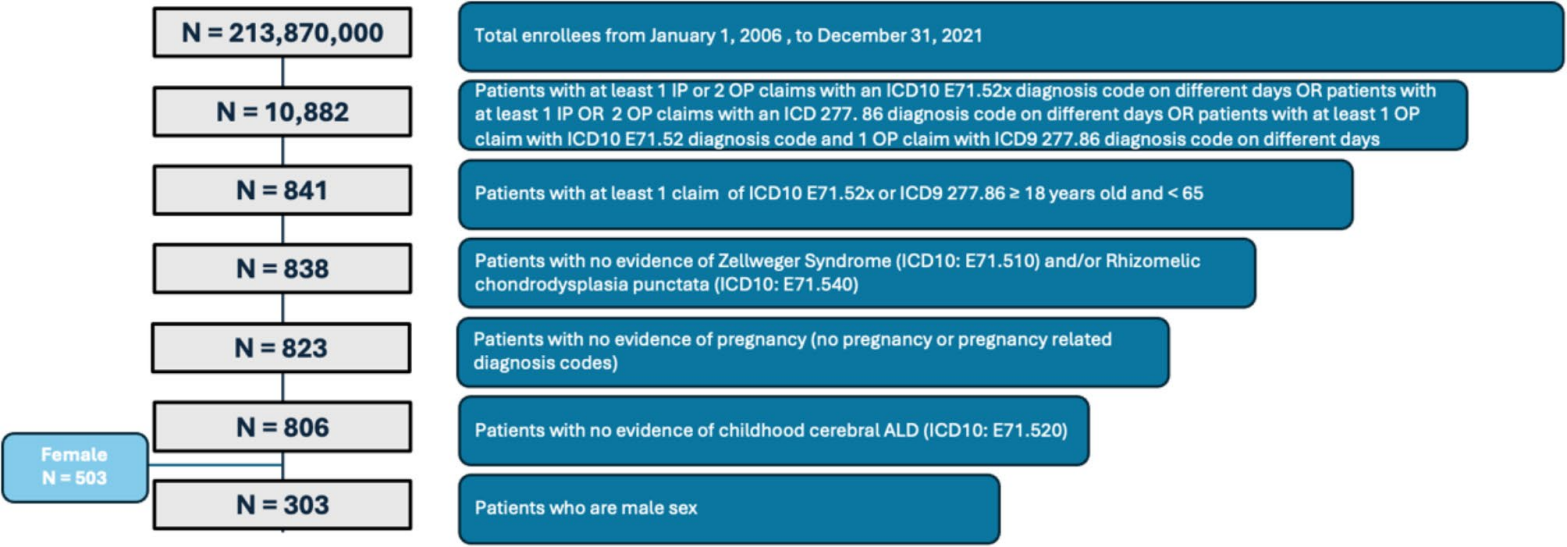
Patient Attrition – inclusion and exclusion criteria

After applying inclusion/exclusion criteria, 303 male patients were identified as probable AMN cases and included in the analysis (Fig. 3). The mean age was 35.1 ± 13.8 8years; more than half (*n* = 171, 56.4%) were aged 18–35 years. A majority resided in the South (*n* = 104, 34.3%) and Midwest (*n* = 92, 30.4%) census regions in the United States. Mean follow-up was 29 ± 27.77 months. Patients with AMN were successfully propensity-matched 1:4 to 1,037 non-AMN controls (Table 4).

**Table 4:** Demographic and clinical characteristics in AMN vs. controls. This denotes the number and proportion of patients with 1 + characteristic during the observation period

Characteristics	Cases (AMN) *N* = 303		Controls (Non-AMN) *N* = 1,037		*P* value
**Demographics**, ***n*(%)**					
Follow-up months, mean ± SD​	29.0 ± 27.7		30.1 ± 25.3		-
Age, mean ± SD​	35.1 ± 13.8		35.2 ± 13.3		-
Age 18–35	171	56.4%	585	56.4%	-
Age 36–51	81	26.7%	275	26.5%	-
Age 52–64	51	16.8%	177	17.1%	-
**Geographic Region**,** n (%)​**					
East	46	15.2%	161	15.5%	-
Midwest	92	30.4%	309	29.8%	-
Other	12	4.0%	69	6.7%	-
South	104	34.3%	326	31.4%	-
West	49	16.2%	172	16.6%	-
**Comorbid Conditions**,** n (%)**					
Charlson Comorbidity index​, mean ± SD​	0.67 ± 1.33		0.19 ± 0.68		< 0.001
Non-Diabetic Neuropathy	33	10.9%	6	0.6%	< 0.001
Hemiplegia or Paraplegia	33	10.9%	< 5	-	< 0.001
Chronic Pulmonary Disease	19	6.3%	27	2.6%	< 0.01
Diabetes	18	5.94%	54	5.2%	0.62
Liver Disease	17	5.6%	8	0.8%	< 0.001
Peripheral Vascular Disease	14	4.6%	9	0.9%	< 0.001
Cerebrovascular Disease	13	4.3%	6	0.6%	< 0.001
Cancer	13	4.3%	14	1.4%	< 0.01
Renal Disease	9	3.0%	6	0.6%	< 0.001
Congestive Heart Failure	7	2.3%	< 5	-	< 0.001
Myocardial Infarction	< 5	-	< 5	-	0.2
Dementia	< 5	-	< 5	-	< 0.01
Rheumatic Disease	< 5	-	< 5	-	0.2
Peptic Ulcer Disease	< 5	-	< 5	-	< 0.01
HIV/AIDs	< 5	-	< 5	-	0.35
Diabetic Neuropathy	< 5	-	6	0.6%	0.19

Measures containing less than 5 patients are masked to protect patient confidentiality

Compared to matched controls, patients with AMN had poorer health status, as measured by the CCI (0.77 ± 1. vs. 0.22 ± 0.77, *p* < 0.001) and more comorbidities. In particular, AMN men had higher rates of peripheral vascular disease (4.6% vs. 0.9%), cerebrovascular disease (4.3% vs. 0.6%), chronic pulmonary disease (6.3% vs. 2.6%), liver disease (5.6% vs. 0.8%), and renal disease (3.0% vs. 0.6%), all *p* < 0.001), as well as higher rates of comorbidities related to AMN vs. controls (hemiplegia/paraplegia: 10.9 vs.<0.6%; non-diabetic neuropathy: 10.9% vs. <0.6%; both *p* < 0.001) (Table 4).

### Healthcare resource utilization

#### Inpatient

**Fig. 4:**
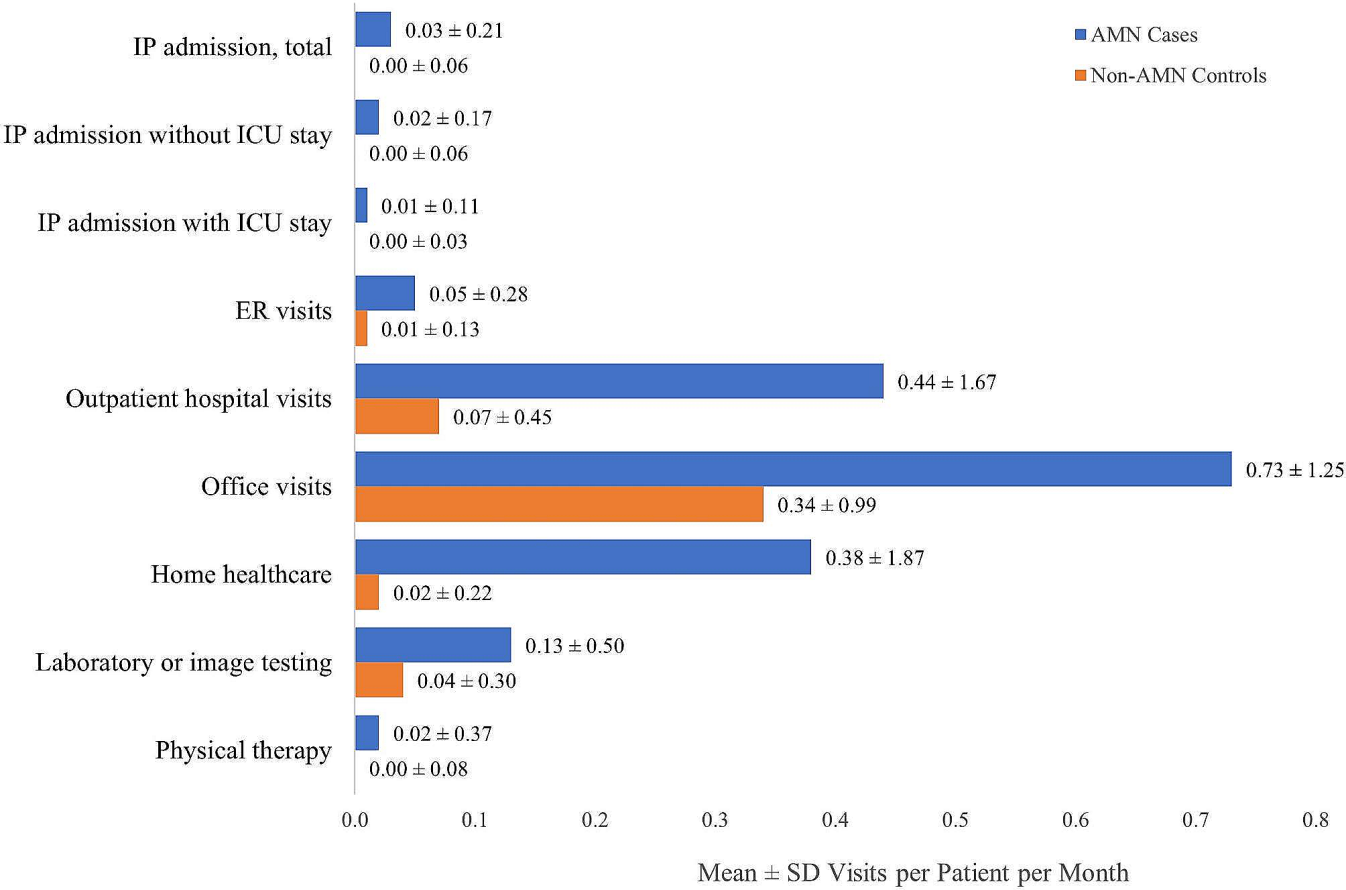
Healthcare resource utilization in patients with AMN vs match controls

Patients with AMN had significantly more inpatient admissions, compared with controls, with 32.0% of patients with AMN having at least 1 inpatient admission with or without an ICU stay (vs. controls: 6.1%; *p* < 0.001). Inpatient admissions with ICU stays, and admissions without ICU stays were also significantly more common, with 13.5% of patients with AMN having at least 1 admission with an ICU stay (vs. controls:1.4%; *p* < 0.001) and 27.7% with at least 1 admission without an ICU stay (vs. controls: 5.6%; *p* < 0.001) (Table 5). Mean length of stay (LOS) for hospitalizations was also longer for patients with AMN, both with an ICU stay (8.7 ± 12.22 vs. 5.1 ± 2.7 days per admission, *p* < 0.05) and without an ICU stay (9.2 ± 15.99 vs. 4.3 ± 2.66 days per admission) (Fig. 4).

**Table 5:** Generic product identifier (GPI) codes

Group	Medication Category	GPI code
Adrenal Insufficiency	Corticosteroids	2,200,000,000
Mood	Anti-depressant	5,800,000,000
	Anti-anxiety	5,700,000,000
	Anti-psychotic	5,900,000,000
	Stimulants	6,100,000,000
Neuropathy	Anti-spasmodic	4,910,000,000
	Anti-convulsant	7,200,000,000
	Analgesic	6,600,000,000; 6,500,000,000; 6,400,000,000
	Musculoskeletal	7,500,000,000
	Neuromuscular	7,400,000,000
Incontinence	Anti-cholinergic	5,020,000,000
	Urinary anti-spasmodic	5,400,000,000; 5,399,200,000
	Genitourinary	5,600,000,000
	Anti-diarrheal	4,700,000,000
	Laxative	4,600,000,000
Sexual Dysfunction	Sex hormones	2,300,000,000; 2,400,000,000; 5,535,000,000
	Genital modulator	4,030,300,000; 2,140,350,000; 4,017,000,000

#### Outpatient services

Outpatient utilization varied by type of service. Nearly all patients with AMN (92.4%) had ≥ 1 office visit (8.8 ± 15.0 encounters PPPY); 52.5% had ≥ 1 laboratory or imaging test (1.6 ± 6.0 encounters PPPY); 33.3% (*n* = 101) had ≥ 1 home healthcare visit (4.6 ± 22.4 encounters PPPY). 17.8% had ≥ 1 claim for DME (0.7 ± 3. encounters PPPY) and 13.9% had ≥ 1 physical therapy visit (0.3 ± 4.4 encounters PPPY). Outpatient utilization was significantly lower in controls, *p* < 0.001 (Fig. 4; Tables 6 and 7).

**Table 6:** Healthcare Resource Utilization in AMN vs. Matched controls. This denotes the number and proportion of patients with 1 + encounter, by type of service

Variables, *n* (%)	Cases (AMN) *N* = 303		Controls (Non-AMN) *N* = 1,037		*P* value
**Inpatient Services**					
**Inpatient admissions**	97	32.0%	63	6.11%	< 0.001
IP without ICU stay	84	27.7%	58	5.66%	< 0.001
IP with ICU stay	41	13.5%	14	1.44%	< 0.001
**Outpatient Services**					
Emergency room (ER) visit	113	37.33%	246	23.7%	< 0.001
Outpatient hospital visit	243	80.2%	432	41.77%	< 0.001
Office visit	280	92.4%	739	71.33%	< 0.001
Home healthcare	101	33.%	86	8.33%	< 0.001
Laboratory or image testing	159	52.55%	370	35.77%	< 0.001
Physical therapy	42	13.99%	34	3.33%	< 0.001
Medications administered in outpatient setting	168	55.5%	283	27.33%	< 0.001
Durable Medical Equipment (DME)	54	17.8%	43	4.22%	< 0.001
Other outpatient	203	67.%	480	46.33%	< 0.001
**Prescription Medications (pharmacy fills)**					
**All Rx**	270	89.%	721	69.5%	< 0.001
**AMN Rx**	250	82.5%	528	50.9%	< 0.001
Adrenal Insufficiency	184	60.7%	246	23.7%	< 0.001
Anti-depressant	78	25.7%	132	12.7%	< 0.001
Anti-anxiety	70	23.1%	93	9.00%	< 0.001
Anti-psychotic	27	8.9%	27	2.6%	< 0.001
Stimulants	17	5.6%	45	4.3%	0.39
Anti-spasmodic	10	3.3%	9	0.99%	< 0.05
Anti-convulsant	77	25.4%	63	6.11%	< 0.001
Analgesic	128	42.2%	361	34.8%	< 0.05
Musculoskeletal	82	27.11%	109	10.5%	< 0.001
Neuromuscular	17	5.6%	8	0.88%	< 0.001
Incontinence	92	30.44%	87	8.44%	< 0.001
Sexual Dysfunction	22	7.33%	19	1.8%	< 0.001

Measures containing less than 5 patients are masked to protect patient confidentiality

**Table 7:** HRU and costs in AMN vs. Matched controls. Costs are shown in USD per patient per year

Costs, mean ± SD	Cases (AMN) *N* = 303	Controls (Non-AMN) *N* = 1,037	Cases (AMN) *N* = 303	Controls (Non-AMN) *N* = 1,037
**Total Costs (Total Inpatient + Total Outpatient + All Rx)**			$29,172 ± $344,643	$2,925 ± $35,110
**Total Costs (Total Inpatient + Total Outpatient + AMN Rx)**			$26,597 ± $343,232	$2,424 ± $29,099
**Inpatient**				
**Total Inpatient** (visits/patient/year)	0.44 ± 2.5	0.04 ± 0.88	$16,697 ± $339,309	$765 ± $9,189
Without ICU stay	0.33 ± 2.11	0.03 ± 0.77	$7,231 ± $113,1911	$523 ± $6,287
With ICU stay	0.1.3	0.01 ± 0.3	$9,466 ± $313,2392	$1241 ± $2,902
Length of stay (LOS) per admission (average, days)	8.88 ± 13.88	4.3 ± 2.44		
LOS without ICU stay	9.22 ± 15.99	4.3 ± 2.66		
LOS with ICU stay	8.77 ± 12.22	5.1 ± 2.7		
**Outpatient**				
**Total Outpatient** (visits/patient/year)			$8,8708 ± $38,208	$1,5435 ± $17,8218
Emergency room (ER) visit	0.5 ± 3.	0.1.6	$340 ± $2630	$829 ± $989
Outpatient hospital visit	5.3 ± 20.00	0.99 ± 5.44	$3,463274 ± $28,163	$2682 ± $8,80180
Office visit	8.88 ± 15.0	4.19.9	$1,309130 ± $3819	$388 ± $4,0650
Home healthcare	4.66 ± 22.4	0.2.6	$1,40540 ± $8802	68$36 ± $428
Laboratory or image testing	1.6.0 ± 6.0	0.55 ± 3.66	$190 ± $2138	59$35 ± $419
Physical therapy	0.33 ± 4.4	0.0.9	$23 ± $405	220$2 ± $20
Medications administered in outpatient setting	3. ± 21.55	0.6.4	$1,30130 ± $15,086	72$57 ± $682
Durable Medical Equipment (DME)	0.7 ± 3.	0.1.4	82$308 ± $6292	$153 ± $183
Other outpatient	4.33 ± 21.66	1.00 ± 5.3	$4774 ± $5550	$138 ± $1,659
**Prescription (pharmacy fills)** *(fills/patient/year)*				
**All Rx**	18.11 ± 23.44	5.44 ± 11.5	$3,8768 ± $18,33033	$725 ± $8,703
**AMN Rx**	12.6 ± 18.5	2.33 ± 7.3	$1,193 ± $4,07907	$224 ± $2,692
Adrenal Insufficiency	4.5 ± 8.11	0.33 ± 2.0	86$118 ± $356	$3 ± $39
Anti-depressant	1.44.5	0.5 ± 2.7	41,420$41 ± $420	$3030 ± $354
Anti-anxiety	0.4 ± 2.55	0.22 ± 1.66	$15 ± $25	$1 ± $13
Anti-psychotic	0.2.2	0.11 ± 0.8	$2707 ± $507	73$7 ± $83
Stimulants	0.2 ± 1.7	0.22 ± 1.66	7$37 ± $46	$3062 ± $362
Anti-spasmodic	0.11 ± 0.88	0.0.2	$34 ± $54	$00 ± $1
Anti-convulsant	1.8 ± 6.0	0.22 ± 1.7	$459 ± $3,0360	$3860 ± $460
Analgesic	1.22 ± 4.6	0.66 ± 3.22	707$70 ± $827	$95 ± $1,1141
Musculoskeletal	1.55 ± 4.4	0.1.3	$6136 ± $1,7047	$18 ± $18
Neuromuscular	0.11 ± 0.99	0.0.2	$50,150 ± $2,950	$02 ± $2
Incontinence	1.11 ± 4.	0.11.1	$1121 ± $705	44$4 ± $44
Sexual Dysfunction	0.22 ± 1.5	0.11.1	$319 ± $499	55$15 ± $175

*LOS per admission w/ICU stay significantly different for cases vs. controls at *p* < 0.05; all other HRU significantly different at *p* < 0.001. Costs for anti-anxiety, anti-psychotic, and analgesic medications not significantly different for cases vs. controls. Costs for inpatient admissions w/ICU significantly different at *p* < 0.05. Costs for all other services significantly different at *p* < 0.001

#### Prescription medications

More patients with AMN used prescription medications, compared with controls (89.1% vs. 69.5%; *p* < 0.001), with a large majority (82.5%; *n* = 250) using prescription medications associated with AMN symptom treatment (vs. controls: 50.9% in controls; *p* < 0.001). The most commonly prescribed medications used by patients with AMN were for adrenal insufficiency (60.7%), pain relief (42.2%), and incontinence (30.4%) (Table 6). Patients with AMN also filled significantly more prescriptions, on average, per patient per year (18.1 ± 23.44 vs. 5.4 ± 11.5 PPPY, *p* < 0.001). Medications for adrenal insufficiency (4.5 ± 8.07), anti-convulsants (1.8 ± 6.0), and musculoskeletal medications (1.5 ± 4.41) were most frequently prescribed. All prescription medication utilization, except for stimulants, was significantly lower in controls, *p* < 0.001 (Fig. 4; Tables 6 and 7).

#### Costs

**Fig. 5:**
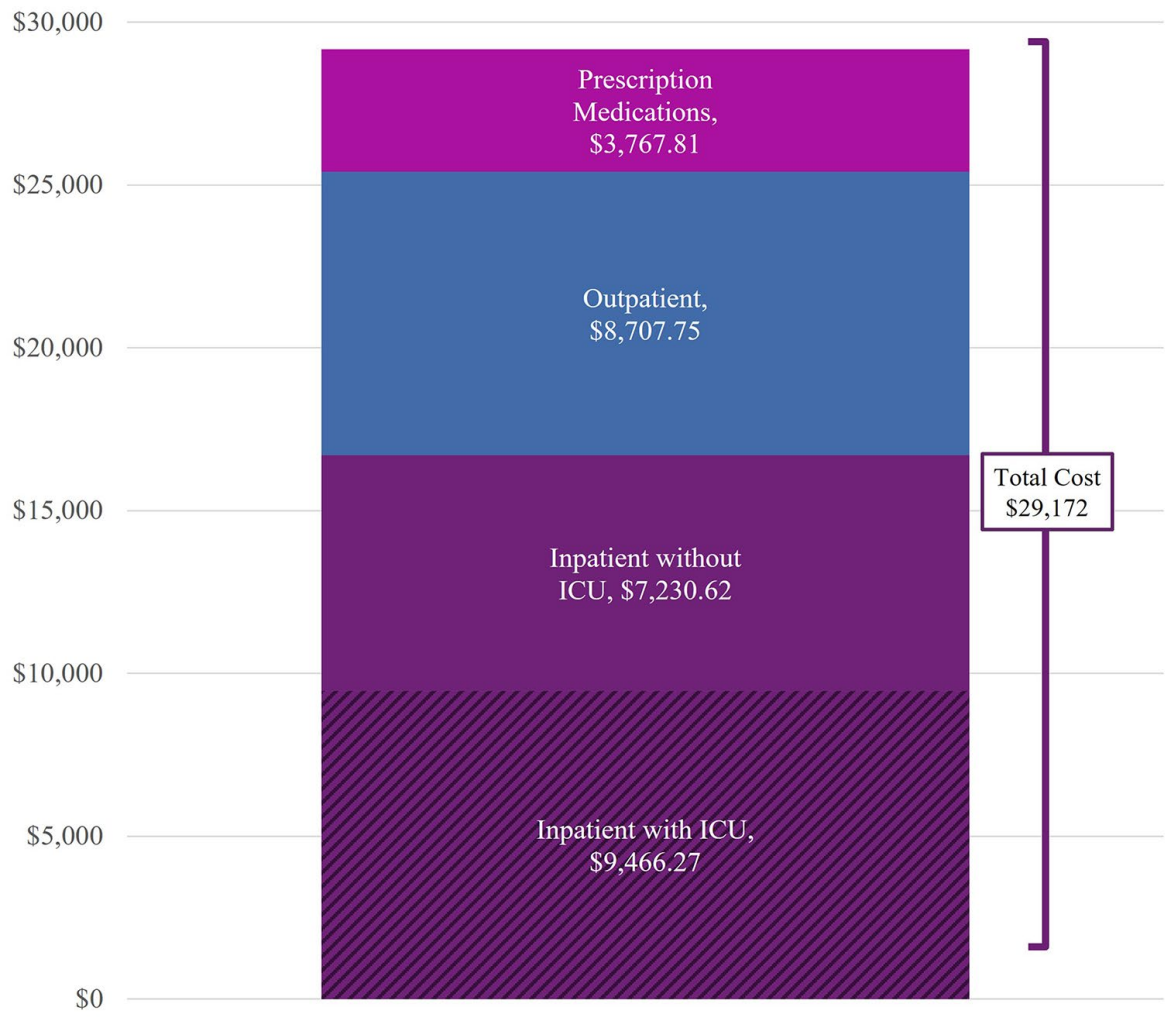
Cost breakdown of care in AMN. Costs are displayed in USD

Healthcare costs paid by commercial payers were significantly greater for patients with AMN, compared with controls. All-cause mean healthcare costs averaged $29,172±$344,643 PPPY for patients with AMN, [controls: $2,926±$35,111, *p* < 0.001]. Inpatient admissions represented 57.2% of these costs (mean $16,697±$339,309 PPPY). Outpatient utilization was associated with 29.8% of all costs PPPY (mean $8,708±$38,208 PPPY). Prescription medications were 12.9% of all costs (mean $3,768 ±$18,033 PPPY) (Fig. 5; Table 7).

### Mortality

After applying study inclusion/exclusion criteria to Medicare LDS enrollment, demographic, and claims data, 242 male patients were identified as probable AMN cases and included in the analysis.

**Fig. 6:**
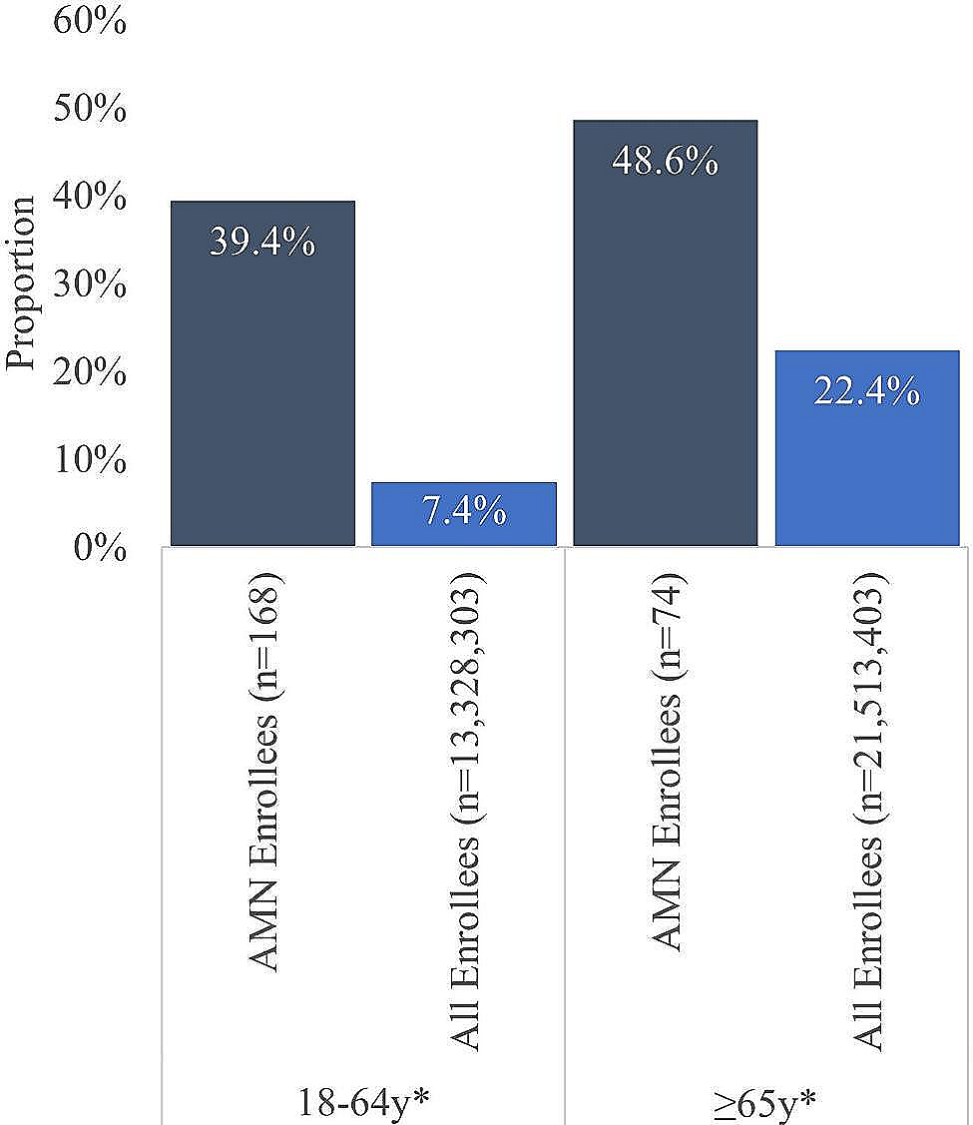
Mortality rate in AMN Medicare enrollees vs all Medicare enrollees

Among Medicare enrollees, mortality rates for men with AMN were significantly higher, compared with all Medicare enrollees. Among disability-eligible Medicare enrollees, men with AMN had 5.3 times higher odds of death compared to all disability-eligible Medicare enrollees, *p* < 0.001. Among age-eligible enrollees, AMN men 2.2 times higher odds of death during the observation period, *p* < 0.001 (Fig. 6).

**Fig. 7:**
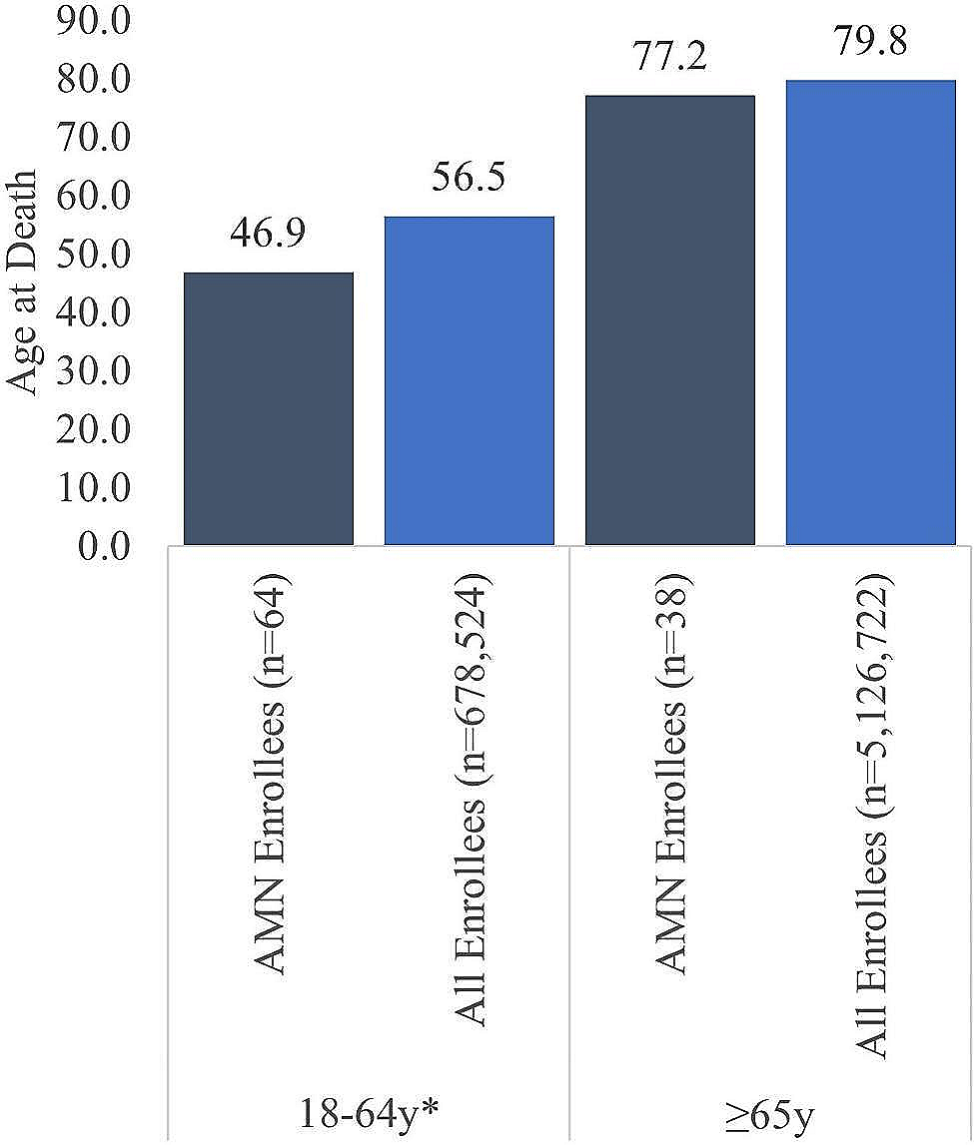
Average age at death for AMN Medicare enrollees who died vs all Medicare enrollees who died. *denotes *p* <0.001

Among male Medicare enrollees who died, those with AMN died at younger ages, compared with other male Medicare enrollees. This effect was particularly pronounced among disability-eligible male Medicare enrollees who died, with mean age at death almost 10 years younger in AMN (46.9 ± 11.3 years vs. controls 56.5 ± 7.8 years; *p* < 0.001), while age-eligible Medicare enrollees who died were younger compared with all enrollees (77.2 ± 10.44 vs. 79.8 ± 8.77 years), but this difference was not significant (Fig. 7).

## Discussion

Our study identifies a previously unrecognized burden of disease in adult men with AMN. Men with AMN and Medicare coverage had significantly higher mortality rates and earlier average age at death. Adult men with AMN had substantially higher healthcare resource utilization and costs, compared with matched controls. We found considerably higher rates of AMN-associated co-morbidities, including pulmonary disease, liver disease, cerebrovascular disease, and cancer. These findings suggest that AMN is associated with a substantially greater healthcare burden than previously appreciated.

To our knowledge, our finding that men with AMN had higher rates of other medical conditions has not been previously noted. It is not known whether the identified comorbidities are related to AMN pathophysiology, complications of AMN and related lifestyle restrictions, or other factors that have not been determined. For example, the observed 5.6% prevalence of liver disease within the cohort could be related to adverse effects of medications used to treat AMN symptoms, or to lifestyle factors. While the etiology is unknown, the findings of increased cardiovascular, pulmonary, renal, and liver comorbidities suggest that there may be previously unrecognized pathophysiology that warrants additional investigation and clinical attention. Given varying degrees of adrenal involvement, AMN is a multi-system disease, and other organ manifestations are not wholly unexpected.

We also found that men with AMN and Medicare coverage died at younger ages and at higher rates compared with all men with Medicare coverage. Among younger, disability-eligible Medicare enrollees, men with AMN died nearly 10 years younger than men without AMN. Differences in age at death among age-eligible male Medicare enrollees with and without AMN were not significant, however, suggesting men with AMN who meet disability-eligibility requirements and enroll in Medicare before age 65 are at an advanced stage of disease – i.e., wheelchair dependent, or with cerebral involvement. While we excluded patients with identifiable cerebral involvement to the best of our ability, this may still be a confounding factor as records of cerebral diagnosis and cause of death were not available Nonetheless, the mortality rate among men with AMN is unusually high compared to other Medicare beneficiaries.

The higher HRU and healthcare costs that we observed in men with AMN may reflect the need to diagnose, monitor, and manage the disease and treat disease related symptoms. 33.3% of patients had home healthcare visits and 17.8% used DME, healthcare services typically not observed in a similarly aged healthy population. The annualized cost of an individual with AMN was $29,172±$344,643 PPPY. These costs, however, are similar to those reported in published studies of other neurological disorders. A study investigating the economic burden of Friedreich’s Ataxia in the US, using patient reported data reported costs related to treating the condition ranged from $8,458-$18,307 PPPY [16]. Furthermore, a 2022 study estimating the economic burden of multiple sclerosis in the US with claims data reported an average excess per-person direct medical cost of $65,612, inclusive of disease-modifying therapy, inpatient and outpatient care, and DME [17]. This also appears comparable, given that expensive biological agents used in multiple sclerosis are unavailable in AMN.

Our study has limitations. The number of commercially insured patients was lower than what would be expected based on the incidence rate of ALD^1^, which may be indicative of underdiagnosis or misdiagnosis of AMN, in part because a specific ICD code for ALD or AMN has been available for only ~ 7 years. It is also possible that the reported values underestimate the AMN disease burden, as our findings were limited by a provider’s utilization of the study’s clinical, procedure, and diagnosis codes of interest recorded in claims data. Notably, there is no ICD-10-CM diagnosis for the adult version of AMN with cerebral involvement and it is possible that the study cohort included individuals with this disease stage, potentially skewing the study’s effect. We did not find evidence consistent with adult cerebral ALD, but we may have missed patients as there is no diagnosis code or other clinical indicator specific to this diagnosis in claims data. Similarly, as the study relies on the diagnostic code used, it is possible that some individuals included may not have met a clinical diagnosis of AMN. The claims data which this study used do not include laboratory values or vital signs. Consequently, it was not possible to confirm AMN with a medical chart review nor identify disease stage. Because this study was conducted in a commercial claims database, the results may not be applicable to those covered by other insurance types. Medications classes were identified using GPI, and not all medications within a class may have been used for management of AMN (Table 8). Finally, we could not determine the reason for prescription medication use in commercially insured adults. Anticonvulsants, for example, may be prescribed for neuropathic pain in AMN, but we could not confirm this, as diagnoses are not recorded on prescription drug claims.

**Table 8:** Healthcare Resource utilization – number and proportion of patients with 1 + utilization during observation

Variables, *n* (%)	Cases (AMN) *N* = 303		Controls (Non-AMN) *N* = 1,037		*P* value
**Inpatient Services**					
Inpatient admissions	97	32.01%	63	6.08%	< 0.001
IP without ICU stay	84	27.72%	58	5.59%	< 0.001
IP with ICU stay	41	13.53%	14	1.35%	< 0.001
**Outpatient Services**					
Emergency room (ER) visit	113	37.29%	246	23.72%	< 0.001
Outpatient hospital visit	243	80.20%	432	41.66%	< 0.001
Office visit	280	92.41%	739	71.26%	< 0.001
Home healthcare	101	33.33%	86	8.29%	< 0.001
Laboratory or image testing	159	52.48%	370	35.68%	< 0.001
Physical therapy	42	13.86%	34	3.28%	< 0.001
Medications administered in outpatient setting	168	55.45%	283	27.29%	< 0.001
Durable Medical Equipment (DME)	54	17.82%	43	4.15%	< 0.001
Other outpatient	203	67.00%	480	46.29%	< 0.001
**Prescription Medications (pharmacy fills)**					
All Rx	270	89.11%	721	69.53%	< 0.001
AMN Rx	250	82.51%	528	50.92%	< 0.001
Adrenal Insufficiency	184	60.73%	246	23.72%	< 0.001
Anti-depressant	78	25.74%	132	12.73%	< 0.001
Anti-anxiety	70	23.10%	93	8.97%	< 0.001
Anti-psychotic	27	8.91%	27	2.60%	< 0.001
Stimulants	17	5.61%	45	4.34%	0.39
Anti-spasmodic	10	3.30%	9	0.87%	< 0.05
Anti-convulsant	77	25.41%	63	6.08%	< 0.001
Analgesic	128	42.24%	361	34.81%	< 0.05
Musculoskeletal	82	27.06%	109	10.51%	< 0.001
Neuromuscular	17	5.61%	8	0.77%	< 0.001
Incontinence	92	30.36%	87	8.39%	< 0.001
Sexual Dysfunction	22	7.26%	19	1.83%	< 0.001

Measures containing fewer than 5 patients are masked to protect patient confidentiality

To our knowledge, this study is the first assessment of the characteristics, healthcare resource use, costs, and mortality in adult patients with AMN. ALD and AMN carry substantial burdens for both the patient and their family, leading to permanent disability and pervasive, difficult-to-manage symptoms that significantly disrupt patients’ quality of life [18, 19]. The prevalence of specific comorbidities, increased odds of mortality, and earlier age at death that we observed are consistent with a high clinical disease burden and poor outcomes. The costs we observed, while considerably higher for AMN compared to non-AMN patients, likely underestimate the true effect of AMN, as they did not account for either direct medical and other costs borne by the patient or effects of AMN on quality-of-life. We also did not measure indirect costs to society, such as loss of working potential and loss of earnings associated with AMN.

Additional research is needed in several areas, including on healthcare costs, HRU and clinical burdens for women with ALD; and male ALD with cerebral inflammatory brain disease, which affects up to 30% of adult AMN men [20]. It is also unclear whether the increased morbidities and mortality are an indirect effect of AMN complications, for example, decreased mobility; or whether they reflect hitherto unrecognized disease complications of ALD. Our results highlight the need to better understand the cause of the higher levels of comorbidity and mortality drivers in AMN to improve clinical care and health outcomes.

### Electronic supplementary material

Below is the link to the electronic supplementary material.

Supplementary Material 1

Supplementary Material 2

## Data Availability

The data used and analyzed in this study are commercially available from IQVIA. [https://www.iqvia.com/locations/united-states/library/fact-sheets/iqvia-pharmetrics-plus]
