# Supplementary material for: Burden of illness and mortality in men with Adrenomyeloneuropathy: a retrospective cohort study
Source: Orphanet J Rare Dis. 2024 Jul 17;19:270. doi: 10.1186/s13023-024-03276-w (PMC11253437; doi:10.1186/s13023-024-03276-w)
Supplement: Supplementary file 1 — Supplementary Material 1 [file 13023_2024_3276_MOESM1_ESM.docx]

# Appendix

| **Table S1. ICD-CM-# ALD + AMN Diagnosis Codes** | | |
| --- | --- | --- |
| **Code Type** | **Code** | **Description** |
| ICD-9-CM | 27786 | Peroxisomal disorders |
| ICD-10-CM | E71521 | Adolescent X-linked adrenoleukodystrophy |
|  | E71522 | Adrenomyeloneuropathy |
|  | E71528 | Other X-linked adrenoleukodystrophy |
|  | E71529 | X-linked adrenoleukodystrophy, unspecified type |

| **Table S2. Charlson Comorbidity Diagnosis Codes** | | | |
| --- | --- | --- | --- |
| **Type** | **Description** | **ICD-9 CM Code(s)** | **ICD-10 CM Code(s)** |
| Charlson Comorbidity | Myocardial Infarction | 410.*, 412.* | I21.*, I22.*, I25.2 |
|  | Congestive Heart Failure | 428.* | I09.9, I11.0, I13.0, I13.2, I25.5, I42.0, I42.5–I42.9, I43.x, I50.x, P29.0 |
|  | Peripheral Vascular Disease | 443.9, 441.*, 785.4, V43.4 Procedure 38.48 | I70.*, I71.*, I73.1, I73.8, I73.9, I77.1, I79.0, I79.2, K55.1, K55.8, K55.9, Z95.8, Z95.9 |
|  | Cerebrovascular Disease | 430.*–438.* | G45.*, G46.*, H34.0, I60.*–I69.* |
|  | Dementia | 290.* | F00.*–F03.*, F05.1, G30.*, G31.1 |
|  | Chronic Pulmonary Disease | 490.*–505.*, 506.4 | I27.8, I27.9, J40.*–J47.*, J60.*–J67.*, J68.4, J70.1, J70.3 |
|  | Rheumatic Disease | 710.0, 710.1, 710.4, 714.0–714.2, 714.81, 725.* | M05.*, M06.*, M31.5, M32.*–M34.*, M35.1, M35.3, M36.0 |
|  | Peptic Ulcer Disease | 531.*–534.* | K25.*–K28.* |
|  | Liver Disease | 571.2, 571.4–571.6, 456.0–456.21, 572.2–572.8 | B18.*, K70.0–K70.3, K70.9, K71.3–K71.5, K71.7, K73.*, K74.*, K76.0, K76.2–K76.4, K76.8, K76.9, Z94.4, I85.0, I85.9, I86.4, I98.2, K70.4, K71.1, K72.1, K72.9, K76.5, K76.6, K76.7 |
|  | Diabetes | 250.0–250.7 | E10-E14 |
|  | Hemiplegia or Paraplegia | 344.1, 342.* | G04.1, G11.4, G80.1, G80.2, G81.*, G82.*, G83.0–G83.4, G83.9 |
|  | Renal Disease | 582.*, 583–583.7, 585.*, 586.*, 588.* | I12.0, I13.1, N03.2–N03.7, N05.2– N05.7, N18.*, N19.*, N25.0, Z49.0– Z49.2, Z94.0, Z99.2 |
|  | Cancer | 140.*–172.*, 174.*.–195.8, 200.*–208.*, 196.*–199.1 | C00.*–C26.*, C30.*–C34.*, C37.*– C41.*, C43.*, C45.*–C58.*, C60.*– C76.*, C81.*–C85.*, C88.*, C90.*–C97.*, C77.*–C80.* |
|  | HIV/AIDS | 042.*–044.* | B20.*–B22.*, B24.* |

| **Table S3. Neuropathy Diagnosis Codes** | | | |
| --- | --- | --- | --- |
| **Condition** | **Code type** | **Code** | **Description** |
| Diabetic Neuropathy | ICD-9-CM | 249.6X | Secondary diabetes mellitus with neurological manifestation |
|  |  | 250.6X | Diabetes with neurological manifestations |
|  | ICD-10-CM | E13.4X | Other specified diabetes mellitus with neurological complications |
|  |  | E11.4X | Type 2 diabetes mellitus with neurological complications |
|  |  | E10.4X | Type 1 diabetes mellitus with neurological complications |
|  |  | E09.4X | Drug or chemical induced diabetes mellitus with neurological complications |
|  |  | E08.4X | Diabetes mellitus due to underlying condition with neurological complications |
| Non-diabetic neuropathy | ICD-9-CM | 337.0X | Idiopathic peripheral autonomic neuropathy |
|  |  | 354.X | Mononeuritis of upper limb and mononeuritis multiplex |
|  |  | 355.X | Mononeuritis of lower limb and unspecified site |
|  |  | 356.X | Hereditary and idiopathic peripheral neuropathy |
|  |  | 357.X | Inflammatory and toxic neuropathy |
|  | ICD-10-CM | G56.X | Mononeuropathies of upper limb |
|  |  | G57.X | Mononeuropathies of lower limb |
|  |  | G58.X | Other mononeuropathies |
|  |  | G60.X | Hereditary and idiopathic neuropathy |
|  |  | G61.X | Inflammatory polyneuropathy |
|  |  | G62.X | Other and unspecified polyneuropathies |
|  |  | G63 | Polyneuropathy in disease classified elsewhere |
|  |  | G90.0X | Idiopathic peripheral autonomic neuropathy |

| **Table S4. Generic Product Identifier (GPI) Codes** | | |
| --- | --- | --- |
| **Group** | **Medication Category** | **GPI code** |
| Adrenal Insufficiency | Corticosteroids | 2200000000 |
| Mood | Anti-depressant | 5800000000 |
|  | Anti-anxiety | 5700000000 |
|  | Anti-psychotic | 5900000000 |
|  | Stimulants | 6100000000 |
| Neuropathy | Anti-spasmodic | 4910000000 |
|  | Anti-convulsant | 7200000000 |
|  | Analgesic | 6600000000; 6500000000; 6400000000 |
|  | Musculoskeletal | 7500000000 |
|  | Neuromuscular | 7400000000 |
| Incontinence | Anti-cholinergic | 5020000000 |
|  | Urinary anti-spasmodic | 5400000000; 5399200000 |
|  | Genitourinary | 5600000000 |
|  | Anti-diarrheal | 4700000000 |
|  | Laxative | 4600000000 |
| Sexual Dysfunction | Sex hormones | 2300000000; 2400000000; 5535000000 |
|  | Genital modulator | 4030300000; 2140350000; 4017000000 |
