# Supplementary material for: Burden of illness and mortality in men with Adrenomyeloneuropathy: a retrospective cohort study
Source: Orphanet J Rare Dis. 2024 Jul 17;19:270. doi: 10.1186/s13023-024-03276-w (PMC11253437; doi:10.1186/s13023-024-03276-w)
Supplement: Supplementary file 2 — Supplementary Material 2 [file 13023_2024_3276_MOESM2_ESM.docx]

| ALD | Adrenoleukodystrophy |
| --- | --- |
| AMN | Adrenomyeloneuropathy |
| CCI | Charlson Comorbidity Index |
| DME | Durable medical equipment |
| ED | Emergency department |
| GPI | Generic Product Identifier |
| HRU | Healthcare resource utilization |
| ICD-9-CM | International Classification of Diseases, 9^th^ revision, Clinical Modification |
| ICD-10-CM | International Classification of Diseases, 10^th^ revision, Clinical Modification |
| ICU | Intensive care unit |
| LDS | Limited Data Set |
| PPPY | Per patient per year |
| PSM | Propensity score matched |
| PT | Physical therapy |
| US | United States |
| USD | United States dollars |
| X-ALD | X-linked adrenoleukodystrophy |
